# Development of a density-based topology optimization of homogenized lattice structures for individualized hip endoprostheses and validation using micro-FE

**DOI:** 10.1038/s41598-024-56327-4

**Published:** 2024-03-08

**Authors:** Patrik Müller, Alexander Synek, Timo Stauß, Carl Steinnagel, Tobias Ehlers, Paul Christoph Gembarski, Dieter Pahr, Roland Lachmayer

**Affiliations:** 1 Institute of Product Development, Leibniz University of Hannover, Garbsen, 30823 Germany; 2 TU Wien, Institute for Lightweight Design and Structural Biomechanics, Vienna, 1060 Austria; 3 Division Biomechanics, Karl Landsteiner University of Health Sciences, Krems, 3500 Austria

**Keywords:** Individualized hip endoprosthesis, Lattice structures, Topology optimization, Micro-FE, Additive manufacturing, Biomedical engineering, Mechanical engineering, Biomedical engineering, Mechanical engineering

## Abstract

Prosthetic implants, particularly hip endoprostheses, often lead to stress shielding because of a mismatch in compliance between the bone and the implant material, adversely affecting the implant’s longevity and effectiveness. Therefore, this work aimed to demonstrate a computationally efficient method for density-based topology optimization of homogenized lattice structures in a patient-specific hip endoprosthesis. Thus, the root mean square error (RMSE) of the stress deviations between the physiological femur model and the optimized total hip arthroplasty (THA) model compared to an unoptimized-THA model could be reduced by 81 % and 66 % in Gruen zone (GZ) 6 and 7. However, the method relies on homogenized finite element (FE) models that only use a simplified representation of the microstructural geometry of the bone and implant. The topology-optimized hip endoprosthesis with graded lattice structures was synthesized using algorithmic design and analyzed in a virtual implanted state using micro-finite element (micro-FE) analysis to validate the optimization method. Homogenized FE and micro-FE models were compared based on averaged von Mises stresses in multiple regions of interest. A strong correlation (CCC > 0.97) was observed, indicating that optimizing homogenized lattice structures yields reliable outcomes. The graded implant was additively manufactured to ensure the topology-optimized result’s feasibility.

## Introduction

Stress shielding is a phenomenon where a stiff metallic implant resists the applied forces instead of the less rigid bone, causing a decrease in mechanical sensory stimuli to the adjacent tissue^1^. This subsequently leads to gradual degradation and loss of bone mass over time. This is a critical challenge in orthopedics, especially in hip arthroplasty, because it reduces bone density, which affects secondary stability and longevity of implants^1–3^. To minimize the effect of stress shielding, the mechanical properties of the implant must be adjusted to closely match the preoperative bone for physiologic force application^4,5^. Additive manufacturing offers the potential to create highly variable topologies, for example, by using lattice structures, which can improve the therapeutic quality of endoprostheses, especially concerning the mentioned problem^6,7^. Due to their adaptable topology of the unit cells used, lattice structures enable an individualized design approach for components by allowing local modification of the mechanical properties^8–10^. In addition, the perforated surface structure inherent in lattice structures promotes bone ingrowth, increasing the endoprosthesis’s secondary stability^11^. However, synthesizing porous structures due to graded lattice structures typically involves substantial computational and modeling efforts. The individualization of these structures is commonly achieved through topology optimization, which is subsequently implemented in complex processes to synthesize the graded lattice structure^12,13^.

Several approaches to the use of lattice structures have already been proposed for the optimization of hip endoprostheses, highlighting the potential of these structures^14–23^. In most cases, the density distribution of the implant is optimized to prevent bone resorption and implant failure at the bone-implant interface. Particularly advanced and promising procedures have recently been presented by Wang et al.^22^ and Garner et al.^19^. Garner et al., and Wang et al., performed multi-objective and multi-constrained topology optimization using a homogenized surrogate model of the lattice structures. This optimization results in a new density distribution of the implant. Subsequently, a finite element (FE) analysis of the density-optimized implant is performed to evaluate it based on criteria such as bone remodeling and interface fracture risk. If necessary, the topology optimization and the analysis process are repeated. The process is iterative until all criteria are met and the optimization converges. The optimized density distribution is used and transferred to a lattice model in the final step. Both studies use asymptotic homogenization to obtain a surrogate mechanical model of the lattice unit cell. Although the homogenized material models of the lattice and the bone are validated, the final optimized implant has not been validated using both the actual lattice microstructure and the microstructure of the bone. Other approaches are similar to both described above but use different optimization criteria, such as the compliance of the implant^14^ or the interface fracture risk^16^. He et al.^14^ optimization objective was the minimization of the volume with the constraint that the compliance of the femur must be within 5 % of the physiological femur in the optimization. The optimization result is additively manufactured, but the final microstructure is not verified. In contrast to the other approaches, Nomura et al.^15^ consider the preoperative stress state in their study by reducing stress shielding through optimization. Furthermore, the bone’s compliance is considered in the optimization process. However, they employ a simplistic bone model involving two homogeneous materials and model the bone with a cylindrical cavity.

Interestingly, based on the current state of the art, there does not yet seem to be an approach to optimize implants that focuses on restoring the preoperative stress state of the bone. In addition, so far, there is no three-dimensional validation of the optimized lattice implant with high-resolution micro-FE ($\mu$FE) models^24,25^, which would take microstructural geometries of the bone and implant into account. Finally, only limited evidence (regarding shape fidelity) suggests that additive manufacturing makes fabricating endoprostheses with topology-optimized structures possible.

Therefore, the objectives of this work were: (1) to present a computationally efficient density-based topology optimization approach for the synthesis of graded lattice structures in hip endoprosthesis that aims to restore the preoperative stress state in the bone, (2) to validate the results using micro-FE models that take the microstructural geometry of the lattice and bone structure into account, (3) to proof the feasibility of manufacturing the optimized implant using additive manufacturing.

## Methology

**Figure 1:**
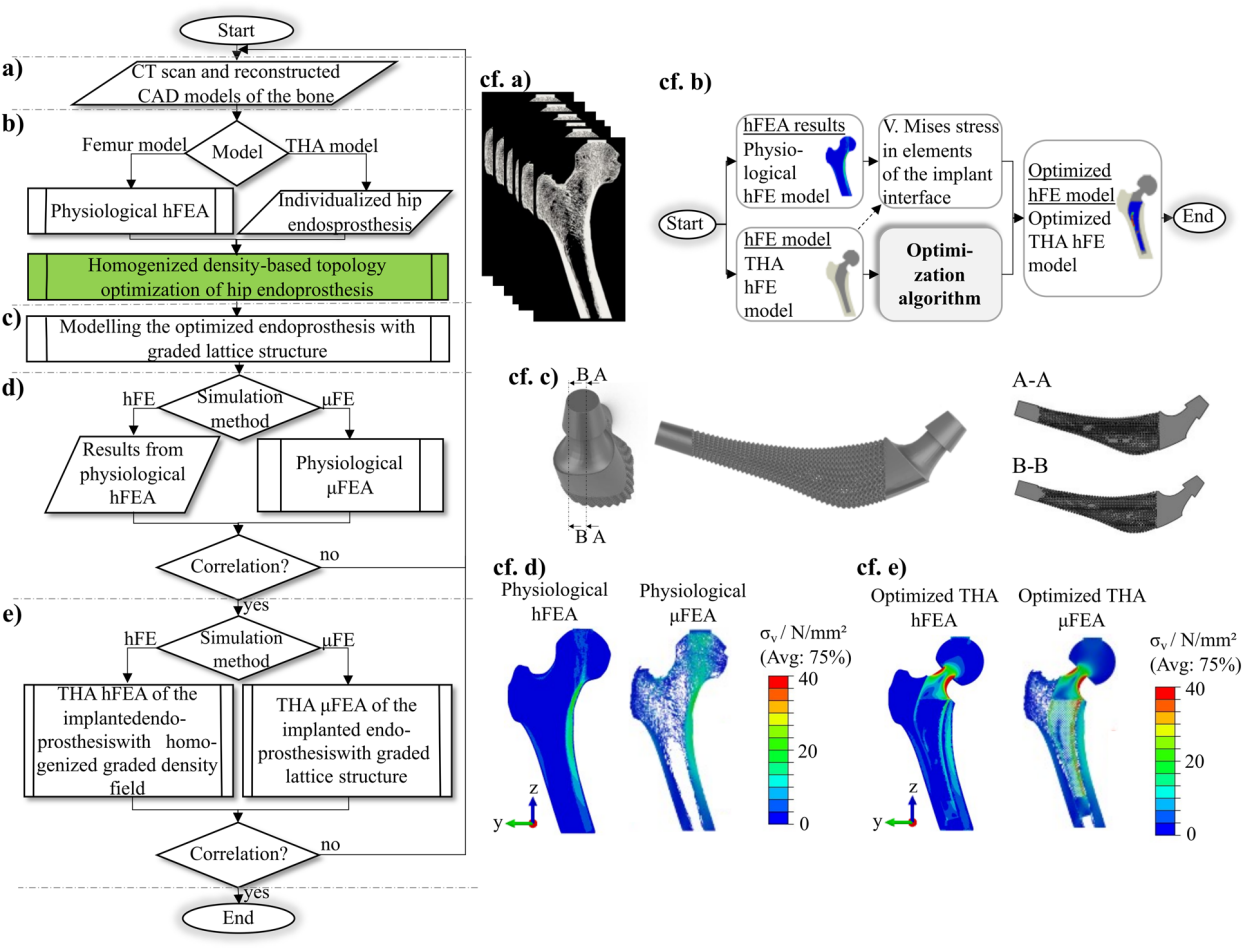
Presentation of the study as a program flow chart with the individual phases: (**a**) selection of the CT scan and reconstruction of the physiological bone, (**b**) the density-based topology optimization of the hip endoprosthesis, (**c**) the synthesis results of the endoprosthesis with graded lattice structure for additive manufacturing, (**d**) the simulation method comparison of the physiological hFE model and physiological $\mu$FE model, and (**e**) the simulation method comparison of the optimized-THA hFE model and optimized-THA $\mu$FE model.

Figure 1 is a graphical overview of the study. First, a human femur’s high-resolution computed tomography (CT) scan (30.3 $\upmu$m resolution) was obtained from a previous study^26,27^. Based on this image, the bone tissue was segmented, and a linear physiological homogenized FE (hFE) model was reconstructed (cf. Fig. 1a).

The density-based topology optimization method then requires two hFE models: first, the physiological hFE model of the femur to perform a static hFE analysis (hFEA) that determines the physiological stress state. In addition, an hFE model of the bone with a virtually implanted individualized endoprosthesis was created, representing the state after total hip arthroplasty (THA), from now on referred to as the THA hFE model. For this purpose, an endoprosthesis with an individualized shape was designed according to Müller et al.^28^. Using these two models, the density-based topology optimization of the hip endoprosthesis in hFE was performed (cf. Fig. 1b). Thus, following the individualization of the implant shape, this study also individualizes the topology, which should maximize the functionality of the implants. This optimization results in a homogenized graded density field of the implant, which attempts to achieve the physiological stress state in the surrounding bone as well as possible in the implanted state. The graded density field was then converted into graded lattice structures of the endoprosthesis (cf. Fig. 1c). In addition, to prove the feasibility of fabricating the lattice structure, the topology-optimized endoprosthesis is manufactured using laser powder bed fusion (LPBF) and then analyzed in a microCT.

Since validation of the implant with lattice microstructures in the hFE model of the bone does not perfectly account for the complexity and heterogeneity of the natural bone tissue, it may lead to inaccurate results. Therefore, validating the implant with lattice and bone microstructures in a THA $\mu$FE model is necessary to confirm the reliability of the results from topology optimization. $\mu$FE analysis ($\mu$FEA) offers the advantage of considering the bone and implant geometry down to the microscale, as the element size in these models is typically well below 100 $\upmu$m. Thus, this approach provides a more accurate representation of actual physical conditions and allows for more precise modeling of the bone structure in conjunction with the lattice structure. Here, the comparison of the simulation methods to validation of the hFE models relative to $\mu$FE models was performed by comparing the stress fields both in the physiological state (without implant) (cf. Fig. 1d) and in the models with optimized-THA (THA hFE model with homogenized graded density field and THA $\mu$FE model with direct graded lattice structures) (cf. Fig. 1e).

### Finite element model meshes, material models and boundary conditions

#### Image processing

A high-resolution CT scan of a human proximal femur (age: 82 years; side: left; sex: female) was taken from a previous study^26,29^. No experiments on human tissue were conducted for this study and no new CT scans of human tissue were acquired; only CT data from a prior study was utilized. For this prior study, the lower limbs of human donors were collected at the Anatomical Institute of the University of Lübeck. The scientific use of human tissue from body donors is permitted by the German law “Gesetz über das Leichen-, Bestattungsund Friedhofswesen des Landes Schleswig-Holstein—Abschnitt II, §9 (Leichenöffnung, anatomisch)” from 04.02.2005. The donors have agreed to the scientific use of their bodies. All methods were carried out in accordance with relevant guidelines and regulations. For a more detailed description of the CT data, the reader is referred to this previous work^29^. In brief, the scan was acquired using an XtremeCT II scanner (Scanco Medical AG, Brüttisellen, Switzerland) and reconstructed with an isotropic voxel size of 30.3 $\upmu$m. The scan was resampled to a voxel size of 90.9 $\upmu$m resolution, rotated such that the femoral shaft was inclined by 20^∘^^30^, cut to a height of 164 mm and segmented using a single level threshold. This high-resolution 3D image was later used to create the voxel-based $\mu$FE models. To create coarsened images for the hFE models, the segmented image was first rescaled such that fully dense bone tissue had a constant grey value of 1200 (representing a bone mineral density of 1200 mg/cm^3^), then three times iteratively blurred using a Gaussian kernel (support: 3; sigma: 0.8), and reduced in resolution by a factor of two, leading to a final resolution of 0.7272 mm for the coarsened image. This image resolution is within the range that can be achieved with a clinical CT scanner^30^.

#### Homogenized FE models

The physiological hFE model is based on a finite element mesh with quadratic tetrahedral elements (C3D10) with an average edge length of 0.5 mm. This model was used for the physiological hFE model representing the physiological condition. This element size was chosen because it showed mesh convergence in a study with tetrahedral elements and represents the finest possible resolution^31^. Furthermore, a THA hFE model of the total hip arthroplasty (THA), encompassing both the femur and the implant, is also a finite element mesh with quadratic tetrahedral elements (C3D10) with an average edge length of 1.7 mm. This size was chosen to increase the computational efficiency of the topology optimization. In this case, the implant and femur interface surfaces were tightly coupled, meaning that no relative motion was possible between the bone and the implant at the interface.

Both hFE models modeled bone material using isotropic, density-dependent material properties. The bone elastic modulus $E_{\text {bone}}$ was computed based on the local density $\rho _{\text {bone}}$ using a power-law with an exponent of two, following previous studies^32,33^:

$$\begin{aligned} E_{\text {bone}} = E_{\text {0, bone}} \cdot \rho _{\text {bone}}^2 \end{aligned}$$

where $E_{\text {0, bone}}$ represents the tissue elastic modulus which was assumed to be 10 GPa in this work^33^, and $\rho _{\text {bone}}$ can be computed from the local greyvalue (*GV*) as $\rho =GV/1200$. The Poisson’s ratio of bone was assumed to be constant and set to $\nu _{\text {bone}} = 0.3$. For the full-density implant as well as the femur head and the embedding, a titanium-based material (Ti6Al4V) is utilized, characterized by an elastic modulus $E_{\text {0, implant}} = 114 \hspace{5.0pt}\text {GPa}$, and a Poisson’s ratio $\nu _{\text {implant}} = 0.3$.

The material model of the graded implant is also described mathematically. Since the stiffness depends on the density of the implant and the density mainly affects the elastic modulus $E_{\text {implant}}$, this relationship must be determined by a density-dependent material law. To represent the material behavior of the density-reduced elements, a body-centered (BC) cell is chosen in this work due to its straightforward geometry, adaptability through parametric adjustments, and compatibility with additive manufacturing techniques without support structures^8^. To determine the mechanical substitute model of the BC unit cell, the elastic modulus is assessed for various densities using the asymptotic homogenization outlined by Dong et al.^34^. Subsequently, the obtained relative densities of the implant $\rho _{\text {r,implant}}$ are interpolated to derive a fourth-degree polynomial that characterizes the mechanical substitute model of the BC unit cell, as depicted by the material equation:

$$\begin{aligned} \begin{aligned} E_{\text {implant}}(\rho _{\text {r,implant}}) =&-80282.34 \cdot (\rho _{\text {r,implant}})^4 + 251060.24 \cdot (\rho _{\text {r,implant}})^3 \\&- 67230.23 \cdot (\rho _{\text {r,implant}})^2 + 10496.82 \cdot (\rho _{\text {r,implant}}) - 22.81. \end{aligned} \end{aligned}$$

All models were simulated with identical boundary conditions to ensure comparability between the simulated models. A load of 1 kN was applied to an embedding located on the femoral head while the distal face of the femur was fixed.

#### Micro-FE models

Voxel-based $\mu$FE models of the physiological bone (without implant) were created by directly converting the segmented high-resolution 3D images (90.9 $\upmu$m voxel size) to a finite element mesh with linear hexahedral elements (C3D8). For the THA $\mu$FE model, the femoral head and the bone material within the implant region were removed, consistent with the THA hFE models. Then, the optimized lattice structure was inserted into the 3D image using image processing, and the modified image was converted into a finite element mesh with hexahedral elements. Note that this means the implant was tightly coupled to the bone, consistently with the hFE models. The number of elements was 92.7 million for the physiological $\mu$FE model and 131.5 million for the THA $\mu$FE model with the optimized implant.

In both $\mu$FE models, isotropic homogeneous material properties were assigned to the bone tissue using the bone tissue elastic modulus $E_{\text {0, bone}} = 10 \hspace{5.0pt}\text {GPa}$ and a Poisson’s ratio $\nu _{\text {bone}} = 0.3$. The implant material was modeled using $E_{\text {0, implant}} = 114 \hspace{5.0pt}\text {GPa}$, and a Poisson’s ratio $\nu _{\text {implant}} = 0.3$ consistently with the hFE models.

As in the hFE models, a load of 1 kN was applied to an embedding located at the femoral head while the distal face of the femur was fixed.

### Density-based topology optimization of hip endoprosthesis with homogenized graded lattice structures using hFEA

The base of this density-based topology optimization are two hFE models. The first model represents the physiological femur of the patient in its preoperative state (physiological hFE model) and contains the target information of the physiological stresses in the bone. The second model represents the femur with the virtually implanted individualized endoprosthesis (THA hFE model) and indicates the actual condition. The optimization goal is to approximate the physiological stresses of the preoperative bone in the postoperative bone of the virtual implanted THA with a topology-optimized density field of the endoprosthesis. To achieve this, it must be possible to represent the physiological stresses in the THA model and, for example, incorporate them as boundary conditions in the model. For this purpose, the interface between implant and bone is detected in the THA hFE model. This is done by searching for all elements in the bone that share nodal points with the implant. The interface-related elements are then sought in the physiological hFE model to capture von Mises stresses. Reducing set elements is necessary considering the 5000 limit for the number of constraints in Abaqus, which applies to stresses from the selected approach. Thus, 4328 uniformly spaced interface elements were chosen, maximizing physiological stress capture within reason. The objective is to approximate these stresses within the bone-implant interface in the optimized-THA hFE model through graded lattice structures by minimizing the energy stiffness measure and the resulting variation of the elastic modulus within the density field of the endoprosthesis.

It is assumed that if the optimized-THA hFE model approximates the correct von Mises stresses in the 4328 elements of the bone-implant interface, the stresses in the rest of the bone will approximate the physiological state to such an extent that the negative effect of stress shielding in the bone is reduced.

#### Topology optimization formulation and design space

The optimization objective of this work was to minimize the energy stiffness measure^35,36^ while satisfying the constraints of preoperative stresses at the implant boundary. The parameters and variables in the following topology optimization are:

- ${{\varvec{P}}}$: The external loading on the system (THA hFE model).
- $\vec {u}$: The corresponding nodal deflections of the loaded nodes.
- $\vec {u_a}$: The prescribed displacements.
- ${{\varvec{R}}}$: The corresponding nodal reaction forces.
- $\rho _{\text {r,implant}}$: The relative density field of the implant, which can be varied between 20% and 100% (design variable).
- $\sigma _{\text {v,physiological-hFEA}}$: The von Mises stresses in physiological bone.
- $\sigma _{\text {v,optimized-THA-hFEA}}$: The von Mises stresses in the optimized-THA model.
- $\mathscr {E}$: A set consisting of 4328 elements at the interface between bone and implant.

The energy stiffness measure describes a stiffness measure without direct physical meaning for dealing with displacement and force boundary conditions in stiffness optimization. It combines two different optimization approaches. First, depending on the boundary conditions, the compliance behaves differently during optimization: if only external forces are applied, the strain energy must be minimized. The optimization objective then results to $$\begin{aligned} min \left( \frac{{{\varvec{P}}}\vec {u}}{2}\right) . \end{aligned}$$ Second, when a load case is driven by prescribed displacements and no external loading is present, the strain energy should be maximized for optimal results. The optimization objective then results with $$\begin{aligned} max\left( \frac{{{\varvec{R}}}\vec {u_a}}{2}\right) . \end{aligned}$$ Abaqus introduces a concept known as the energy stiffness measure to combine both boundary conditions in the objective function. This approach allows the stresses from the physiological femur $\sigma _{\text {v,physiological-hFEA}}$ as a constraint to grade the density field of the THA hFE model of the implant in such a way that it approximates the physiological stress state in the virtually implanted and optimized state $\sigma _{\text {v,optimized-THA-hFEA}}$ to reduce stress shielding. Accordingly, the formulation of density-based topology optimization for this work results in:

$$\begin{aligned} \text {minimize} \quad&\left( \frac{{{\varvec{P}}}\vec {u}}{2}\right) - \left( \frac{{{\varvec{R}}}\vec {u_a}}{2}\right) \\ \text {subject to} \quad&\sigma _{\text {v,optimized-THA-hFE}, e} = \sigma _{\text {v,physiological-hFE}, e} \quad \forall e \in \mathscr {E} \\&0.20 \le \rho _{ \text {r,implant}, i} < 1.00 \quad \forall i \end{aligned}$$

Note that in contrast to conventional stiffness optimization that needs a mass constraint to avoid a full density solution, this optimization approach only utilizes the energy stiffness measure and the von Mises stresses at the bone-implant interface. This approach inherently leads to a reduction in implant density as the stresses at the interface in an unoptimized (full density) implant are typically lower than in the physiological femur^1^. Therefore, implant density will be reduced during the optimization to reach higher stresses at the bone-implant interface that are closer to the physiological loading state.

**Figure 2:**
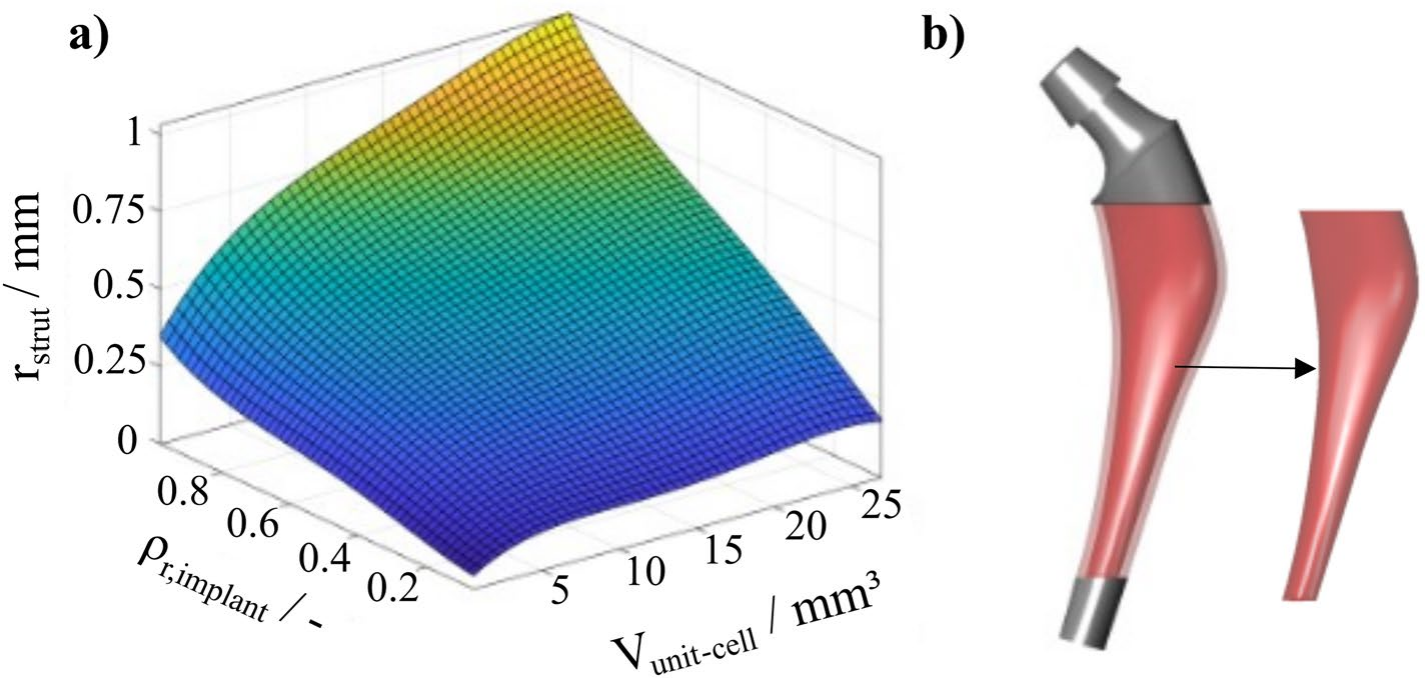
Design space of topology optimization: (**a**) representation of the radius of the strut as a function of relative density and unit cell volume, (**b**) only the inner region of the implant is topology optimized for stability reasons and filled with graded lattice structures.

The only design variable of the topology optimization was the relative density of the implant $\rho _{\text {r,implant},i}$. The lower and upper bounds of $\rho _{\text {r,implant},i}$ were defined as 20 and 100%, respectively. These values were chosen because they are without restrictions, producible by additive manufacturing for the selected unit cell size (a relative density of 20 % results in a strut radius $r_{\text {Strut}}$ of 0.175 mm for a constant unit cell volume ($V_{\text {unit-cell}}$) of 2 $\times$ 2 $\times$ 2 $\textrm{mm}^{3}$ (cf. Fig. 2a), which does not contradict the manufacturing restrictions of the LPBF system used^37^). The focus of optimization is solely on the density of the hFE model of the implant, which leads to local variations of the elastic modulus (cf. Fig. 2). Moreover, the implant is divided into four regions: the lower and upper segments are intentionally not optimized. This design decision was made to ensure the structural integrity of the implant, as optimization of these areas could affect the implant’s overall stability and functionality (also during hammering-in). Lattice structures are applied only in the middle area, where the marginal layer (a one-unit cell layer) has a constant density of 50 % to ensure high bone ingrowth. The layer has unit cell sizes from 2 $\times$ 2 $\times$ 2 $\textrm{mm}^{3}$ to 3 $\times$ 3 $\times$ 2 $\textrm{mm}^{3}$ with corresponding $r_{\text {Strut}}$ from 0.175 to 0.22 mm. This configuration results in 0.5–0.8 mm pore diameters in the perforated surface, which, according to various studies, maximizes bone ingrowth^38^. Thus, only the inner region is topologically optimized using this approach and is the design space (cf. Fig. 2b).

#### Criteria for the evaluation of the density-based topology optimization

As the optimization goal was to approximate the stress state in the THA model to the physiological femur, the stress deviation $\Delta$ is introduced, which is based on the discrete stress distributions in the physiological hFE model $\sigma _{\text {v,physiological-hFEA}}$, the unoptimized-THA hFE model $\sigma _{\text {v,unoptimized-THA-hFEA}}$ (implant without density grading) and the optimized-THA hFE model $\sigma _{v,\text {optimized-THA-hFEA}}$. This parameter methodically shows analogies to the calculation of the Stress Shielding Increase^39^. For this purpose, the stresses of each element in the bone of the model before ($\sigma _{v,\text {unoptimized-THA-hFEA}, e}$) and after optimization ($\sigma _{\text {v,optimized-THA-hFEA}, e}$) are subtracted from the physiological stress state ($\sigma _{\text {v,physiological-hFEA}, e}$):

$$\begin{aligned} \Delta _{\text {THA-hFEA}, e} = \sigma _{\text {v,unoptimized-THA-hFEA}, e} - \sigma _{\text {v,physiological-hFEA}, e} \end{aligned}$$

and

$$\begin{aligned} \Delta _{\text {optimized-THA-hFEA}, e} = \sigma _{\text {v,optimized-THA-hFEA}, e} - \sigma _{\text {v,physiological-hFEA}, e}. \end{aligned}$$

However, there are limits to the information that can be gained from the averaged stress deviations alone, so a local assessment is also performed. This is done by creating point clouds that color code the stress deviation. The relationship is that the closer $\Delta _{\text {optimized-THA-hFEA}}$ is to 0, the better the optimization worked.

In addition, two boxes with respective cubic dimensions of 5 $\times$ 5 $\times$ 5 $\textrm{mm}^{3}$ are formed in the Gruen zones (GZ) 1 (lower limit) and 7 (upper limit), which are strongly affected by stress shielding^40^ and two additional ones in GZ 3 and 6. The GZ are specific regions around the femur, identified for assessing the interface between the femoral stem of an endoprosthesis and the bone, crucial for evaluating prosthesis stability and bone remodeling^41^. Subsequently, all von Mises stresses are averaged therein and visualized using box plots to illustrate the statistical distribution of the averaged von Mises stresses in the boxes. Furthermore, to evaluate the model accuracy in these GZ, the root mean square error (RMSE) of the stress deviations between the physiological femur model and optimized total hip arthroplasty (THA) model in comparison to an and unoptimized-THA model is used as a measure of the deviations between the different models. The RMSE places more emphasis on outliers and statistically evident stress deviations, enhancing the robustness of the results. Thus, conclusions about stress shielding can be drawn by interpreting the RMSE.

### Synthesis of the optimized endoprosthesis with graded lattice structure

**Figure 3:**
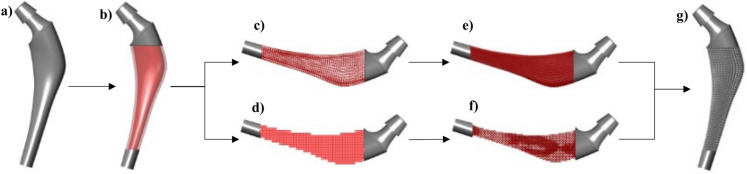
The synthesis process of the implant with a graded lattice structure, starting from (**a**) the individualized endoprosthesis as a complete implant, (**b**) the definition of the design space with the inner area, which is optimized, and the outer edge area, which is filled with lattice structures of a constant density. Subsequently, the volumes are filled with unit cell boxes, (**c**) as a grid of unit cells of the outer edge area and (**d**) as a cube representation of unit cells of the inner area, and in (**e**)–(**f**) the unit cells are filled with struts as well as thickened. In the last step (**g**), the volumes are combined, and the topology-optimized implant is finalized.

A generic algorithm is applied to synthesize the lattice structures, which is fully automated and can be used for every implant geometry. This procedure is visually scripted in Rhino (plugin: Grasshopper) and based on own preliminary work. The synthesis procedure is demonstrated in Fig. 3a–g.

The design space of the implant that will be filled with graded lattice structures is divided into inner and outer regions. Homogeneous 2 $\times$ 2 $\times$ 2 $\textrm{mm}^{3}$ unit cells and the optimized density field are employed for the inner area. In contrast, the outer edge area is filled with unit cells with a constant density; the strut radius is computed based on the relative density and volume of the unit cell. A relative density of 0.2 corresponds to $r_{{\text {strut}}}$ = 0.175 mm, and a relative density of 1 corresponds to $r_{{\text {strut}}}$ = 0.85 mm (resulting in a fully fused unit cell). There is a linear relationship between these boundaries.

Subsequently, the unit cells are filled with line representations of the struts representing the BC unit cell, enabling mathematical manipulation. This allows for efficient adaptation of the lattice structures with minimal computational effort (such as achieving a smooth transition between the inner and outer lattice structures). After these processing steps, the relative density for each unit cell was calculated based on the density-based topology optimization results. Finally, in the last step, the struts are thickened according to the relative density to reproduce the optimized distribution from the density-based topology optimization and their corresponding elastic modulus values accurately. The functions also facilitate a seamless transition between unit cells with different densities.

### Feasibility of manufacturing the optimized endoprosthesis with graded lattice structure

The hip endoprosthesis was manufactured from stainless steel (1.4404) using the LPBF process. This process was carried out on an EOSINT M280 machine, a state-of-the-art system. The EOSINT M280 offers a build volume of 250 $\times$ 250 $\times$ 325 mm, with a resolution range of 30–50 $\upmu$m. The machine has advanced monitoring and control systems, ensuring consistent quality and repeatability. After fabrication, only the support structures were mechanically removed, and the component was cleaned.

Samples were then cut out from the implant, scanned in a microCT (Bruker microCT, SkyScan 1275), manually searched for flaws, and the geometric error of the additive manufacturing was analyzed by a nominal-actual comparison with the CAD model.

### Micro-FE validation of the homogenized density-based topology optimization

To validate the result of the homogenized density-based topology optimization by $\mu$FEA, a comparison between the simulation methods (hFE and $\mu$FE) was performed. Both the physiological and the model with the optimized topology optimized-THA were considered in the comparison.

The metric for validation was von Mises stress, averaged in cubic region of interest (ROI) with 5 mm side length. This size was chosen as it was reported to be the lower bound for determination of apparent elastic material properties of trabecular bone^42,43^. 100 ROIs were used and selected randomly across the femoral bone volume. Boxes that were not filled to at least 90 % are deleted (resulting in fewer ROIs than 100). This is especially the case with THA models, as the implant bed is empty, and thus, the volume inside is not filled.

The correlation between the two simulation methods was then analyzed by Lin’s concordance correlation coefficient (CCC) according to Lin^44^, using the averaged von Mises stresses in all ROIs. Lin’s CCC quantifies both correlation and agreement between two data sets. The level of agreement is determined according to McBride^45^ by calculating the 95 % confidence interval (one-tailed) and taking the lower border. Thereby, a lower border corresponds to > 0.99 near perfection, > 0.95–0.99 substantial, > 0.90–0.95 moderate, and $\le$ 0.90 poor.

In addition, the “goodness of fit” with the coefficient of determination ($R^{2}$) is evaluated^46^. $R^{2}$ is a statistical ratio used to measure how well the observed data from the micro-FE model fit the predicted values of the hFE model. It is a measure of how well the model explains the variability. The $R^{2}$ value ranges from 0 to 1. $R^{2}$ = 0 indicates that the model doesn’t explain any of the variations in the data and doesn’t fit at all. $R^{2}$ close to 0 suggests that the model explains only a tiny portion of the data’s variations. $R^{2}$ close to 1 signifies that the model explains most of the data’s variations very well; an $R^{2}$ of 1 would mean a perfect fit of the model to the data.

### Hardware and software

The CT image processing was performed in medtool 4.5 (Dr. Pahr Ingenieurs e.U., Pfaffstätten, Austria). The coarsened CT image of the femur was utilized to generate an FE mesh using Materialise Mimics v.23.0 and 3-matic v.15.0. The individualized model of the hip endoprosthesis and subsequently, the graded lattice structure was synthesized in Rhino 7 v.7.33.23213.13001 (Plugin: Grasshopper). The physiological hFE model and the homogenized density-based topology optimization were solved with 12 cores from 3.7 to 4.5 GHz. The physiological hFE model was solved in an average CPU time of 6 min, and the homogenized density-based topology optimization in 23.5 h using Abaqus v.6.25 and the optimization package Tosca Structure R2021x Build R423. The $\mu$FE models were solved with 20 cores with 3.6 GHz using ParOSol^47,48^. The physiological model was solved in 8.4 h, and the THA model was solved in 7.8 h. The nominal-actual comparison between synthesized and additively manufactured implants was performed with the Zeiss Quality Suite v4.1.1318.0.

## Results

### Density-based topology optimization of hip endoprosthesis with homogenized graded lattice structures

**Figure 4:**
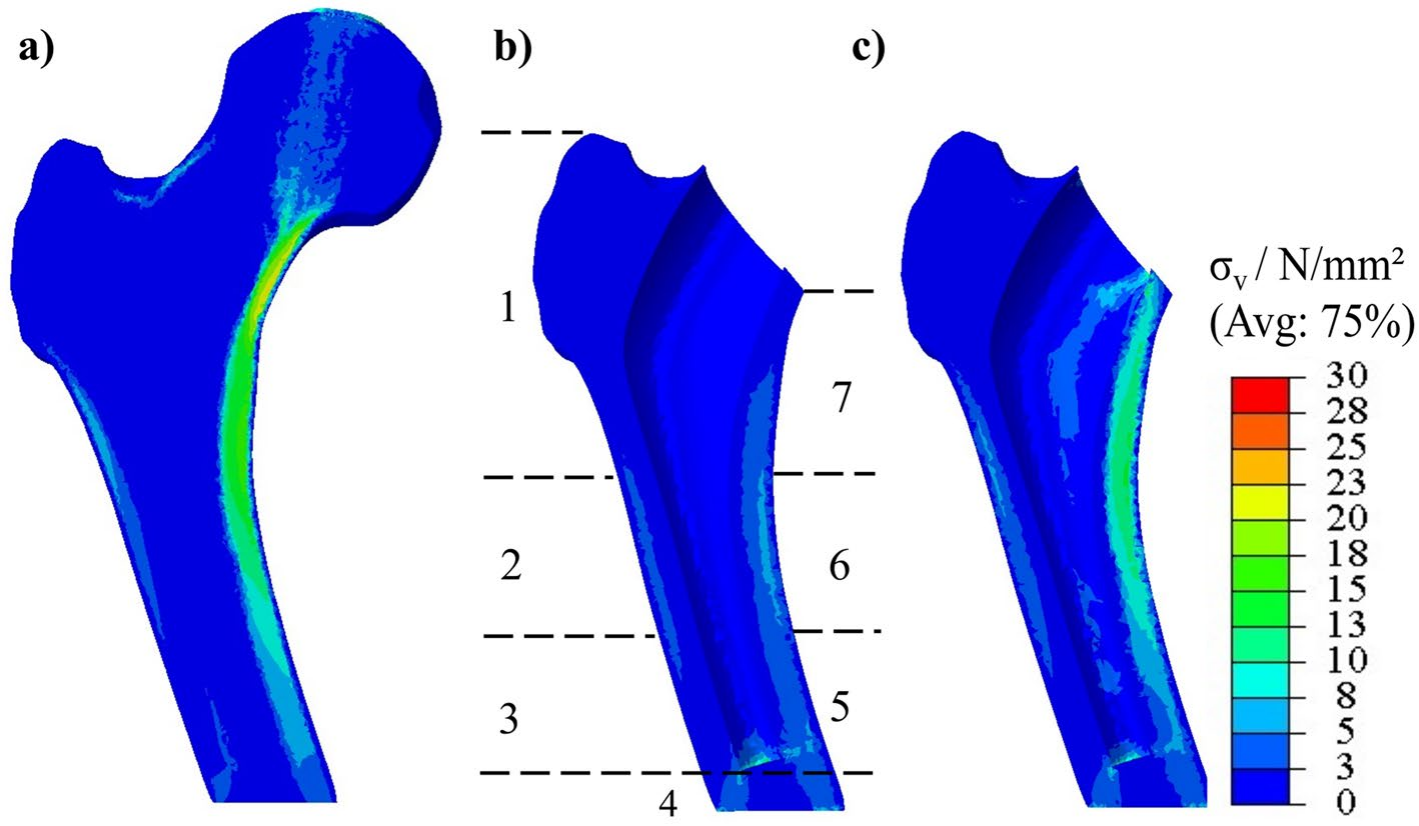
Stresses in the different femur models: (**a**) physiological hFE model, (**b**) unoptimized-THA hFE model (with implant bed and corresponding GZ), (**c**) optimized-THA hFE model (with implant bed).

Figure 4 shows the resulting von Mises stress of a hFEA in the physiological, unoptimized-THA and optimized-THA hFE model.

The differences were most pronounced in the medial side of the femur (GZ 6, 7). In the unoptimized-THA hFE model stresses are also reduced in the diaphysis (GZ 1, 2) (cf. Fig. 4b). In the physiological hFE model, stresses reach up to 20 N/mm^2^ but decrease to approximately 5–10 N/mm^2^ in the unoptimized-THA hFE model. At the same time, stress increases postoperatively in the diaphysis below the implant (GZ 3, 4). The stresses increase here from about 5 to 10 N/mm^2^. Moreover, the stresses in the physiological hFE model also exist in the distal diaphysis. Preoperatively these are about 10 N/mm^2^, postoperatively under 5 N/mm^2^ (cf. Fig. 4a and b). At the same time, in Fig. 4c, the optimized-THA hFE model shows an increase of the von Mises stress, especially in the proximal metaphysis (GZ 6, 7) in comparison to the unoptimized-THA model. Here, the stresses are again at a qualitatively similar level as in the physiological bone model, which indicates that the optimization was successful.

**Figure 5:**
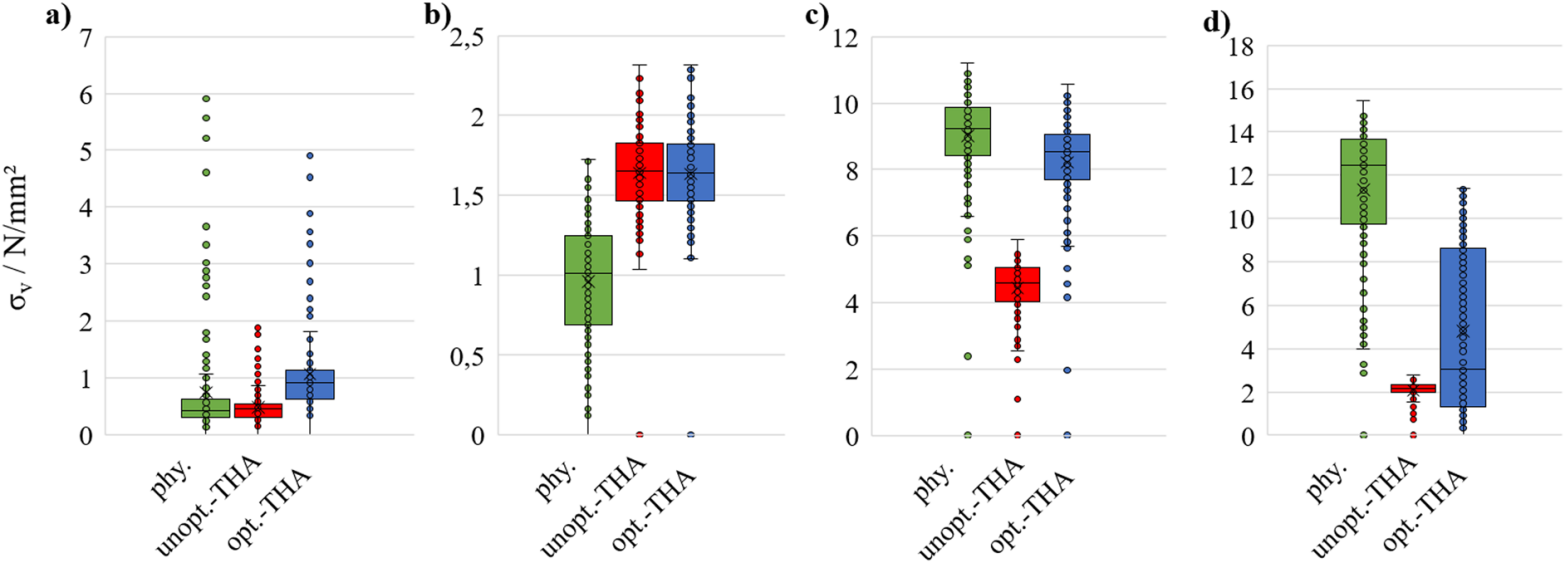
Boxplot representation for the von Mises stresses of the physiological hFE model, unoptimized-THA hFE model, optimized-THA hFE model in the GZ (**a**) 1, (**b**) 3, (**c**) 6, (**d**) 7.

Figure 5a–d visualizes the boxplots in the relevant GZ 1, 3, 6, and 7 for all three hFE models.

Especially in the areas with higher stresses in the physiological state, an approximation of the optimized-THA state is evident, whereas the unoptimized-THA state lowers all stresses except for the one in GZ 3 (and 4), which is typical for hip arthroplasty. This is confirmed above all by the von Mises stresses in GZ 6 and 7. The physiological stresses in GZ 6 are, on average, approx. 9.2 N/mm^2^, in the unoptimized-THA at 4.7 N/mm^2^ and in the optimized-THA again at 8.9 N/mm^2^. In GZ 7, the variance of the result is evidently larger, so the stresses between the physiological hFE model and optimized-THA hFE model do not agree sufficiently well here. Nevertheless, an improvement over the unoptimized-THA hFE model is evident as the stresses return to the physiological range. In regions where the physiological hFE model already displays low stresses (averaging less than 2 N/mm^2^), the impact of the optimization is less pronounced. However, an uptick in stresses is still observed in GZ 1 within the optimized-THA hFE model.

**Figure 6:**
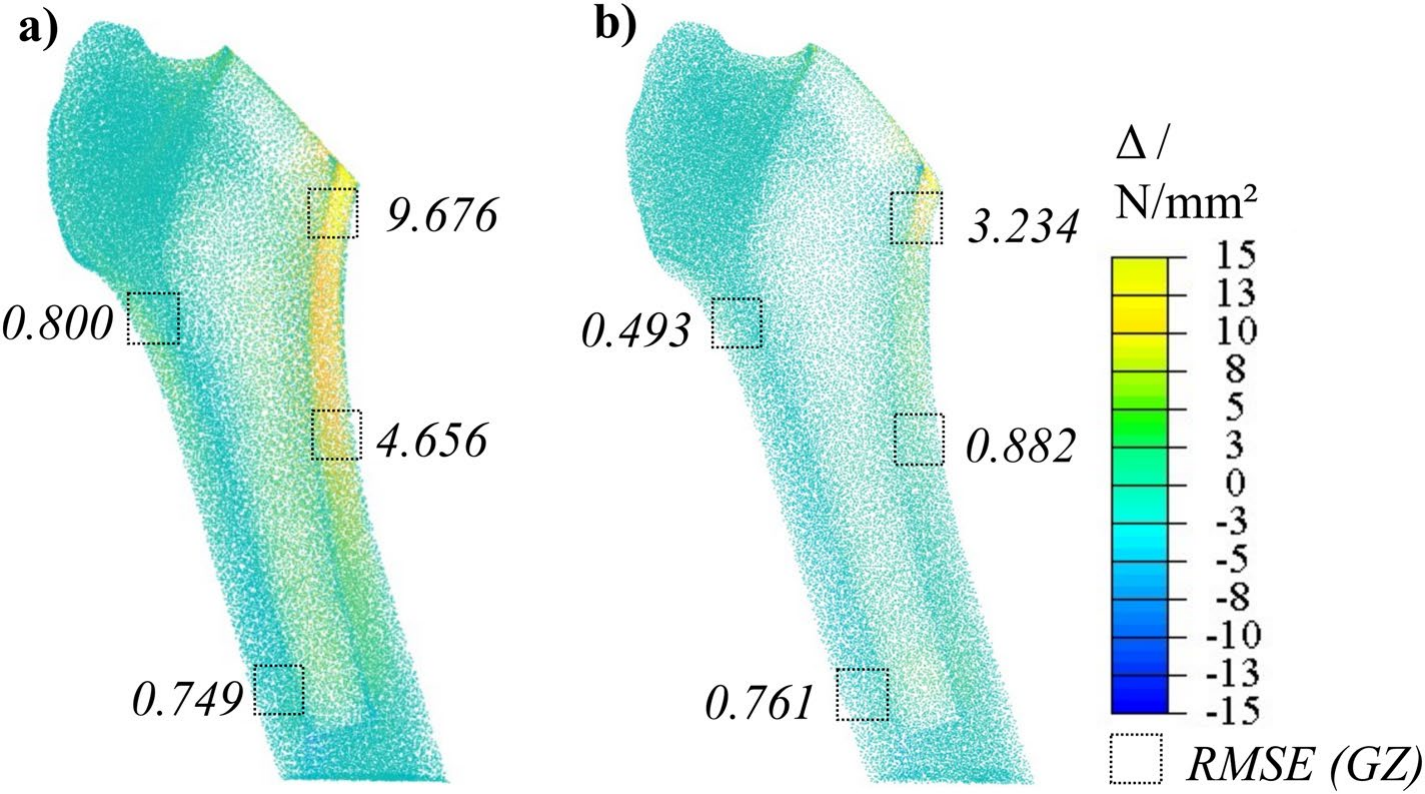
Stress deviations in the postoperative condition between (**a**) $\Delta _{\text {THA-hFE}}$ and (**b**) $\Delta _{\text {optimized-THA-hFE}}$ with corresponding RMSE values of the respective GZ.

Figure 6 shows the visualization of the deviations of the von Mises stresses for the endoprosthesis and the RMSE values for the relevant GZ in the (a) unoptimized- and (b) optimized-THA hFE model. It can be seen that the largest deviations from the femur are in the region of the medial margin of the medial metaphysis (GZ 6, 7). Laterally, the span of stress deviation is narrower. Nevertheless, both areas experience deviations reaching up to 15 N/mm^2^, but these are distinctly confined in spatial extent within the optimized-THA hFE model. The deviations in metaphysis up to 15 N/mm^2^ were almost completely reduced to below 1 N/mm^2^. The RMSE values confirm these findings. While the RMSE in the unoptimized-THA hFE model is high, especially in GZ 6 and 7, in the optimized-THA hFE model, it is reduced by about 81 % in GZ 6 and by about 66 % in GZ 7. In GZ 1, the error is reduced by about 39 %, and in GZ 3 it increased marginally. Globally, the mean $\Delta$ was reduced by 54 % from 1.36 N/mm^2^ to 0.61 N/mm^2^.

### Synthesis of the optimized endoprosthesis with graded lattice structure and feasibility of manufacturing

**Figure 7:**
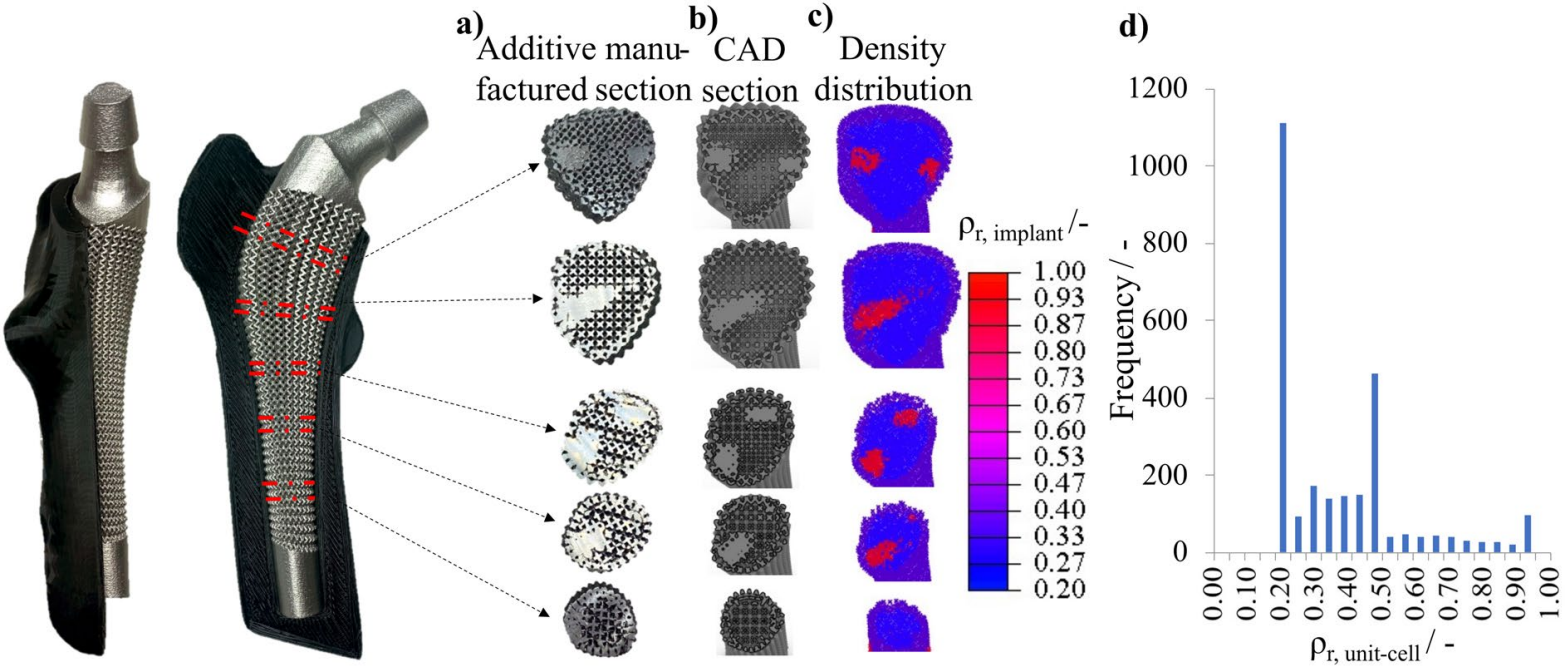
The result of the endoprosthesis with graded lattice structures in five section views as (**a**) an additive manufactured model, (**b**) CAD model, (**c**) density distribution of the topology optimization, and (**d**) histogram of the relative densities.

The endoprosthesis with graded lattice structure is shown in Fig. 7 in different representations (in different views and sectional planes of (a) additive manufacturing of the implant, (b) the CAD model, as well as (c) the result of the density-based topology optimization as a density-based point cloud). To synthesize the graded lattice structure in the optimized endoprosthesis, the relative densities from the topology optimization are used to vary the strut radius ($r_{{\text {strut}}}$) of the unit cells. Figure 7d represents a histogram of the relative densities that occur.

**Figure 8:**
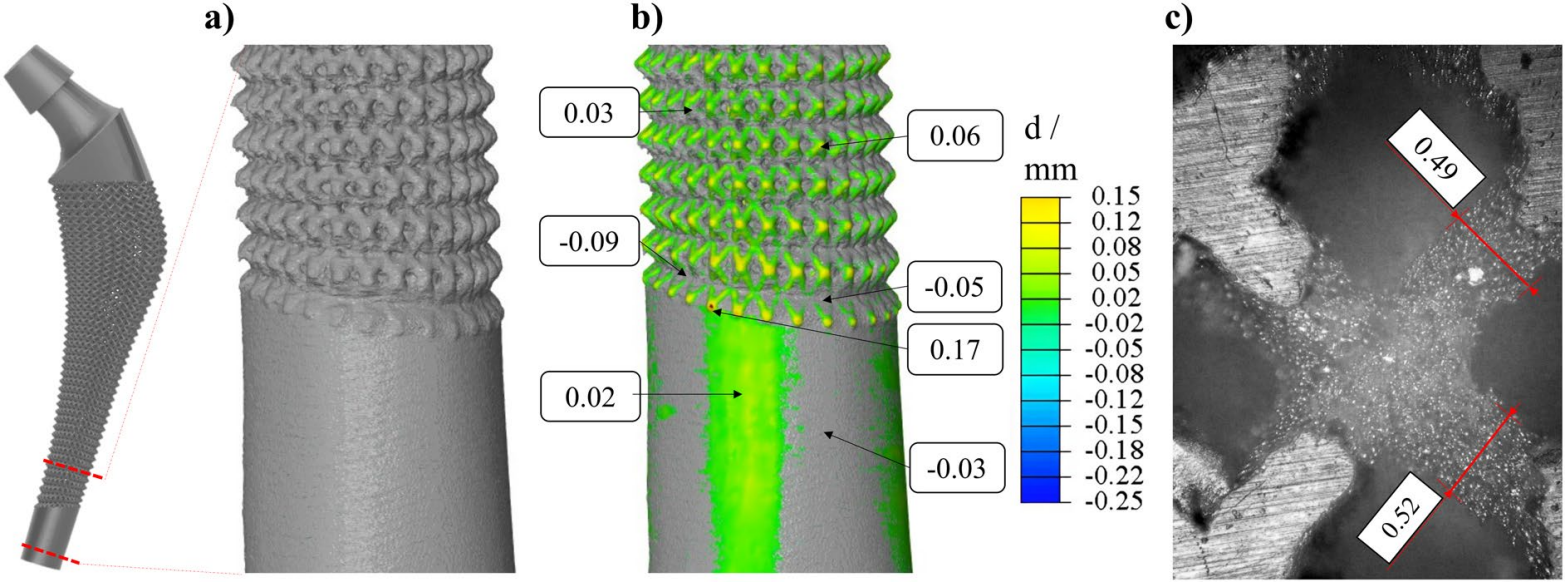
Verification of the additive manufacturing quality in the lower part of the endoprosthesis as (**a**) reconstruction of the microCT scan, (**b**) nominal-actual comparison between real part (gray) and CAD model (color), and (**c**) an optical microscope image to evaluate the shape fidelity of the inner struts.

In addition to the visual verification of the endoprosthesis between the CAD model, the point cloud of the density field, and the additively manufactured demonstrator in the sectional planes, a nominal-actual comparison is performed. For this purpose, the lower part of the endoprosthesis (this area was chosen because the available microCT has sample size restrictions, as well as the thinnest area, had to be selected to ensure radiography with as few artifacts as possible (cf. Fig. 8a). When comparing the CAD model and the CT-scanned microstructure, the surface distance was average between +/− 0.03 mm in the investigated region. The extreme values here were a deviation of − 0.09 and + 0.17 mm (it should be noted that this may be an artifact from the CT scan, as the stainless steel has a high density and is prone to artifact formation) (cf. Fig. 8b). To be able to evaluate the additive manufacturing inside the design space, an image was taken with an optical light microscope, which confirms the shape fidelity of the additive manufacturing process inside as well (cf. Fig. 8c). The comparison with an optical light microscope showed that the strut diameter deviated less than 0.02 mm (compared to the target strut diameter of 0.5 mm).

### Micro-FE validation of the physiological model

**Figure 9:**
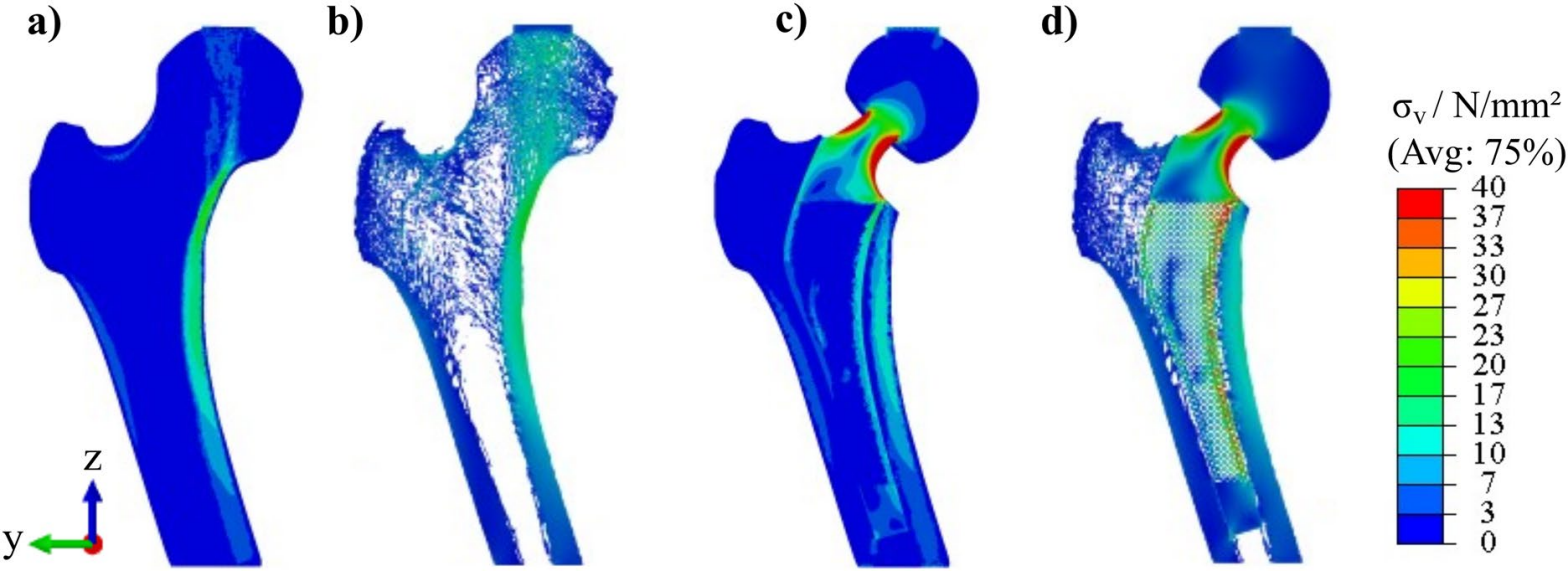
Results of the different simulation methods, (**a**) physiological hFEA, (**b**) physiological $\mu$FEA, (**c**) optimized-THA hFEA, (**d**) optimized-THA $\mu$FEA.

Figure 9a, b illustrates the comparison of the simulation methods for the physiological model using cross-sectional images obtained from respective hFEA and $\mu$FEA. These images depict the distribution of von Mises stresses.

**Figure 10:**
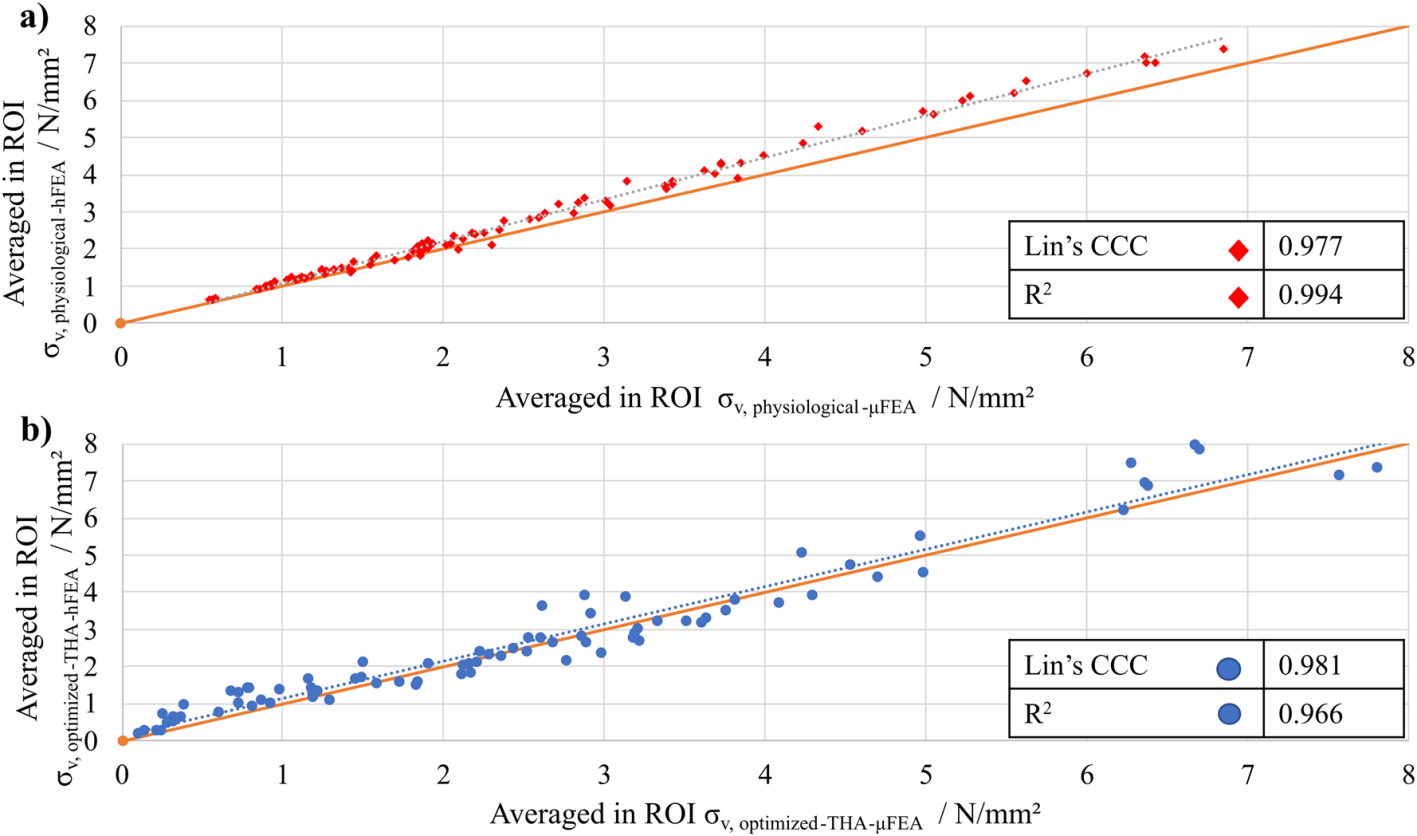
Correlation analysis and calculation of Lin’s CCC to validate simulation results in (**a**) physiological hFEA and $\mu$FEA and in (**b**) with optimized-THA hFEA and $\mu$FEA.

Lin’s Coefficient of Concordance was calculated to be 0.977, suggesting a substantial correlation between the averaged von Mises stress values in hFEA and $\mu$FEA of the physiological models. This high CCC value indicates a strong agreement, suggesting a consistent relationship between the two datasets (cf. Fig. 10a). Additionally, the coefficient of determination was computed as 0.994, further confirming the strong linear relationship between the variables. Given that the lower border of the one-tail confidence interval is 0.970, the level of agreement is rated as substantial. An analysis of the sensitivity of the number of ROIs can be found in Supplementary Material S1 to validate the appropriateness of the selected ROIs.

### Micro-FE validation of the optimized-THA model

Figure 9c, d shows the difference between hFEA and $\mu$FEA using cross-sectional images derived from the optimized-THA model. The images show the von Mises stress distribution.

Lin’s Coefficient of Concordance in 88 ROIs (starting from 100 randomly selected ROIs and the subsequent deletion of ROIs that are not full) was calculated to be 0.981, suggesting a substantial correlation between the averaged von Mises stress values in hFEA and $\mu$FEA with optimized-THA models. This high CCC value indicates a strong agreement, suggesting a consistent relationship between the two datasets (cf. Fig. 10b). Additionally, the coefficient of determination was computed as 0.966, further confirming the strong linear relationship between the variables.

Given that the lower border of the one-tail confidence interval is 0.971, the level of agreement is rated as substantial.

## Discussion

This work demonstrates that density-based topology optimization in a homogenized THA model can evidently reduce the effects of stress shielding. This reduction is achieved by establishing a specific external stress state in the bone by grading a density field within the optimized endoprosthesis. This proof of concept was demonstrated with a simulative validation of a high-resolution CT scan and the direct model of the graded lattice structure in the design space of the implant in a three-dimensional $\mu$FEA. Finally, the manufacturability of the graded lattice structures by additive manufacturing was also proven.

Regarding objective (1) of this work, the density-based topology optimization in homogenized FE models presented here proves to be equally effective compared to other studies. Owing to the varied evaluation criteria employed across the comparative studies, the analysis of the optimization results from this work remains qualitative in nature. While Garner et al.^19^ reduce bone remodeling by 64 %, Wang et al.^22^ describe a reduced bone loss at about 42 %. He et al.^14^ can minimize stress shielding by more than 50 %, and Nomura et al.^15^ increase stresses by about 32 % with the optimized implant. All studies identified the most evident effect of stress shielding in the medial metaphysis (GZ 6, 7), and their results are based on a comparison with an unoptimized or generic implant. In this work, a reduction of the RMSE between the stress deviation of the physiological hFE model and unoptimized-THA hFE model and the stress deviation of the physiological hFE model and optimized-THA hFE model by 81 % (GZ 6) and 66 % (GZ 7) was calculated. Stress deviations were reduced globally by 54 %.

Regarding objective (2), this work suggests that homogenized FE models are suitable for density-based topology optimization despite the simplified material representation of the lattice and bone microstructure. This result is in line with Garner et al.^19^, who used a high-detail 2D FE model that captures the lattice microstructure (but not the bone microstructure) to validate the bone remodeling and interface fracture risk of homogenized FE models of hip implants with optimized lattice structures. The results of this study extend the findings of Garner et al.^19^ using 3D micro-FE models that also include the bone microstructure for validation. Good agreement between homogenized and micro-FE models has also been reported previously for intact bones and conventional bone-implant systems. Pahr et al.^49^ compared homogenized and micro-FE model predicted stiffness of 12 vertebral bodies and found CCC values ranging from 0.37 to 0.97 depending on the homogenized material modeling strategy. Synek et al.^50^ compared peri-implant strain energy densities in homogenized and micro-FE models of 15 screw-bone constructs and found CCC values from 0.77 to 0.96. Despite the good correlation of the homogenized FE models with micro-FE models, and despite the good agreement of micro-FE models of bone-implant systems with experimental results reported in the literature (e.g., see Steiner et al.^51^), an experimental validation of a hip implant with an optimized lattice structure in a real bone remains to be performed.

The proof of the feasibility of the synthesized endoprosthesis, which was objective (3), has been also explored by Nomura et al., Wang et al., and He et al. However, it is worth noting that these studies do not include a direct comparison with the CAD model or any further investigations in this specific aspect of verification. As such, no conclusive comparison can be made at this point. However, studies from other domains also show that the lattice structure’s print quality and shape fidelity align with the state of the art^52^. Since the implant was printed only from a stainless steel alloy (1.4404) and not from the simulated titanium alloy (Ti6Al4V), a comparison with studies describing both the printing and dimensional fidelity of 1.4404 and Ti6Al4V is necessary. Various studies demonstrate the comparability of print quality between the materials^53,54^. The results from the nominal-actual comparison show averaged deviations of +/− 0.02–0.09 mm (0.17 mm in one exceptional case) between the additive manufactured model and the CAD model, which demonstrates the good shape fidelity and agrees with the findings from the literature^54^.

The research showcased in this work has limitations that necessitate further investigations. A limitation of this study is its reliance on a single CT scan and one endoprosthesis, which could constrain the findings’ generalizability. Another limitation is that a perfect bone-implant interface was assumed in both models (hFE models and $\mu$FE models). Additionally, to evaluate bone remodeling in a more defined way in the future, a direct optimization should be performed concerning a parameter describing bone remodeling, such as the stress shielding increase (SSI)^39^ or the Bone Remodeling parameter according to Huiskes and Weinans^55^. Thus, a stress state could be targeted to promote bone remodeling in specific areas and maximize bone ingrowth. Furthermore, interface failure is not considered further since overloading is assumed to be excluded if the physiological stress state is reached in the interface layer. However, since damage can still occur, future consideration is necessary. Furthermore, no failure criterion of the lattice structures is considered in the optimization, which theoretically can lead to stresses above the yield strength of the implant material. Another limitation is the lack of consideration of the fatigue strength of the optimized and additively manufactured lattice structures. Various studies have investigated the fatigue strength of additively manufactured lattice structures of the same material (Ti6Al4V) with different densities and cell types^56–58^. These studies have shown that the occurring stresses, the cell type used, and the grading significantly influence fatigue strength. The stresses observed in this study under the selected boundary conditions can be expected to provide sufficient fatigue strength for various lattice structures tested (cf. Fig. 9). However, further investigations are essential to verify exactly this application. For example, investigations based on ISO 7206-4, which provides a test method for the fatigue behavior of hip stems, are necessary to investigate these structures.

## Conclusion

The main objective of the work was to present a method for density-based topology optimization of prosthetic implants, which is highly computationally efficient by simulating homogenized models of the bone and the implant. The topology optimization reduced the stress deviation between the unoptimized-THA hFE model and the optimized-THA hFE model, evidently reducing the effect of stress shielding. Subsequently, the results obtained by the hFE models were validated by comparison to $\mu$FE models that take the bone and lattice microstructure geometry into account (CCC > 0.97), providing evidence that hFE models are indeed suitable for efficient topology optimization of the hip endoprosthesis and provide reliable results. Manufacturability was confirmed using additive manufacturing techniques, with a qualitative comparison assessing the optimized implant geometry’s production effectiveness. Future research should address the identified limitations to enhance the outcomes and gain a deeper understanding of the impact of stress shielding on patient well-being. Making these necessary adjustments allows for improved results and a more refined understanding of this area.

### Supplementary Information

Supplementary Information.

## Data Availability

The datasets generated during and/or analysed during the current study are available from the corresponding author on reasonable request.
