# Supplementary material for: Development of a density-based topology optimization of homogenized lattice structures for individualized hip endoprostheses and validation using micro-FE
Source: Sci Rep. 2024 Mar 8;14:5719. doi: 10.1038/s41598-024-56327-4 (PMC10923877; doi:10.1038/s41598-024-56327-4)
Supplement: Supplementary file 1 — Supplementary Information. [file 41598_2024_56327_MOESM1_ESM.pdf]

Analysis of sensitivity of the number of ROIs of the subsection „Micro-FE validation of the physiological model“

To confirm the validity of the number of ROIs, a study with 50 ROIs was performed for the physiological hFE and µFE models to ensure that the variability between models can be adequately represented with several ROIs < 100 (Fig. A1). It is important to note that only bone stresses and not implant stresses were used, even if the ROI in the boundary layer of the THA model theoretically intersects the implant.

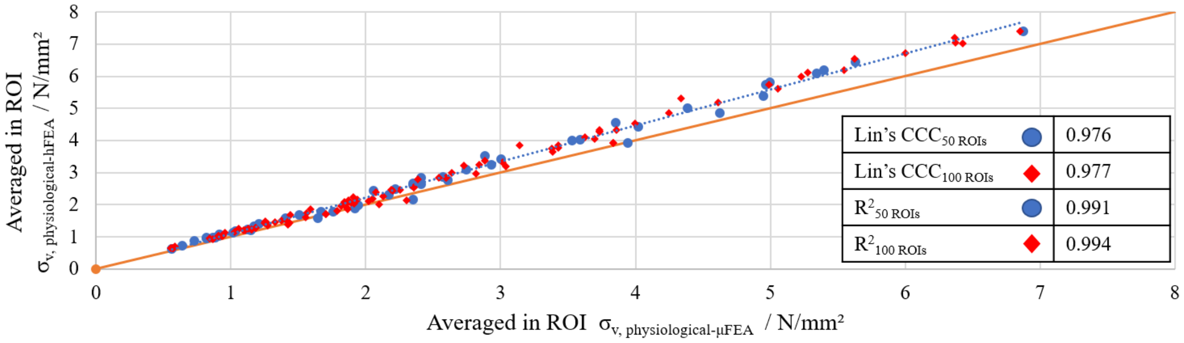

Figure A1: Correlation analysis and calculation of Lin's CCC for 50 and 100 ROIs to show the sensitivities of the study.

Lin's Coefficient of Concordance in 50 ROIs was calculated to be 0.976, suggesting a substantial correlation between the averaged von Mises stress values in hFEA and µFEA of the physiological models. To confirm the study's variability and validate the selection of the 50 ROIs, the same analysis was performed again with 100 ROIs. The results (Lin's CCC = 0.997 and $R^2 = 0.994$) confirm that the variability between models can be adequately represented with several ROIs < 100.
